# Fish and Mammalian Phagocytes Differentially Regulate Pro-Inflammatory and Homeostatic Responses *In Vivo*

**DOI:** 10.1371/journal.pone.0047070

**Published:** 2012-10-23

**Authors:** Aja M. Rieger, Jeffrey D. Konowalchuk, Leon Grayfer, Barbara A. Katzenback, Jeffrey J. Havixbeck, Moira D. Kiemele, Miodrag Belosevic, Daniel R. Barreda

**Affiliations:** 1 Department of Biological Sciences, University of Alberta, Edmonton, Alberta, Canada; 2 School of Public Health, University of Alberta, Edmonton, Alberta, Canada; 3 Department of Agriculture, Forestry and Nutritional Sciences, University of Alberta, Edmonton, Alberta, Canada; Charité-University Medicine Berlin, Germany

## Abstract

Phagocytosis is a cellular mechanism that is important to the early induction of antimicrobial responses and the regulation of adaptive immunity. At an inflammatory site, phagocytes serve as central regulators for both pro-inflammatory and homeostatic anti-inflammatory processes. However, it remains unclear if this is a recent evolutionary development or whether the capacity to balance between these two seemingly contradictory processes is a feature already displayed in lower vertebrates. In this study, we used murine (C57BL/6) and teleost fish (*C. auratus*) *in vitro* and *in vivo* models to assess the evolutionary conservation of this dichotomy at a site of inflammation. At the level of the macrophage, we found that teleost fish already displayed divergent pro-inflammatory and homeostatic responses following internalization of zymosan or apoptotic bodies, respectively, and that these were consistent with those of mice. However, fish and mice displayed significant differences *in vivo* with regards to the level of responsiveness to zymosan and apoptotic bodies, the identity of infiltrating leukocytes, their rate of infiltration, and the kinetics and strength of resulting antimicrobial responses. Unlike macrophages, significant differences were identified between teleost and murine neutrophilic responses. We report for the first time that activated murine, but not teleost neutrophils, possess the capacity to internalize apoptotic bodies. This internalization translates into reduction of neutrophil ROS production. This may play an important part in the recently identified anti-inflammatory activity that mammalian neutrophils display during the resolution phase of inflammation. Our observations are consistent with continued honing of inflammatory control mechanisms from fish to mammals, and provide added insights into the evolutionary path that has resulted in the integrated, multilayered responses that are characteristic of higher vertebrates.

## Introduction

Inflammation is a complex and highly regulated response to pathogen infiltration and tissue damage. While this response is critical to pathogen clearance, inflammatory processes can lead to substantial tissue injury if unchecked [1]–[3]. Thus, there has been a significant drive for co-evolution of regulatory programs to minimize deleterious effects on surrounding tissues [4].

As a mechanism to control tissue damage, inflammation-associated apoptosis triggers anti-inflammatory programs in mammals [4]. *In vitro* studies have shown that phagocytosis of apoptotic cells drives a decrease in pro-inflammatory antimicrobial killing mechanisms [5]–[7]. *In vivo*, apoptotic bodies reduce thioglycollate-induced leukocyte infiltration and promote the resolution of thioglycollate or LPS-driven inflammation [8], [9]. Further, *in vitro* and *in vivo* studies have shown that the clearance of apoptotic cells within an inflammatory site also promotes the production of anti-inflammatory immune mediators, notably interleukin (IL)-10, transforming growth factor (TGF)-β, and platelet activating factor, as well as a decrease in pro-inflammatory cytokines, including tumor necrosis factor (TNF)-α, IL-6 IL-8, IL-12, IL-17, IL-23, prostaglandin E2, leukotriene C4 and thromboxane B2 [10]–[17]. Among others, the shift in the balance between TNF-α and TGF-β contributes to the ‘quenching’ of reactive oxygen and nitrogen species [18]. Removal of the oxidative stress associated with ROS production favors effective uptake of apoptotic cells and further promotes anti-inflammatory programs [19], [20]. Importantly, although continued ROS production can lead to unnecessary tissue damage, premature neutrophilic apoptosis, and inhibition of apoptotic cell uptake, NADPH oxidase also appears to play an important role for the effective resolution of inflammation, by contributing to macrophage recognition and clearance of activated neutrophils at sites of inflammation [21].

Phagocytes contribute to both pro-inflammatory and anti-inflammatory (resolution) responses at infectious foci [11], [20]–[23]. However, it remains unclear if this dichotomy is a recent evolutionary development or whether the capacity to balance between these two seemingly contradictory processes following phagocytosis is a feature already displayed by lower vertebrate phagocytes. Recent evolutionary developments may have provided novel strategies for the control of inflammation, but may contribute to the development of inflammation-associated diseases as new mechanisms look to integrate into existing immune regulatory networks. Importantly, phagocytes are known to internalize pathogens, apoptotic bodies or both at sites of inflammation, raising questions about the contributions of individual phagocytes to the inflammatory process *in vivo*.

In this study, we examined the effects of homeostatic phagocytosis on the regulation of pro-inflammatory antimicrobial responses. Using a comparative model of zymosan-induced peritonitis, the impact of apoptotic bodies on reactive oxygen species (ROS) production was measured *in vivo* in both mice and fish. Zymosan is a commonly used pathogen mimic that induces self-resolving peritonitis *in vivo* [24]–[28]. Previous *in vitro* and *in vivo* studies have shown that zymosan phagocytosis results in activation of pro-inflammatory responses that include induction of pro-inflammatory cytokines, production of reactive oxygen and nitrogen intermediates and increased infiltration of leukocytes, predominantly neutrophils [28]–[34]. Our findings suggest that the divergent responses to pathogenic vs. homeostatic particles at the level of the macrophage are already well established in lower vertebrates. However, notable differences were found between lower and higher vertebrates in the cellular components, kinetics, and magnitude of the inflammatory response. Our results provide added examples for the increased compartmentalization of cellular responses across leukocyte subsets in higher vertebrates. Most notably, our results suggest that the newly identified neutrophilic contributions to the down-regulation of acute inflammatory processes may represent a recent evolutionary development. This continued honing of phagocytic responses is likely a testament to the ever-increasing role of the phagocyte in the promotion and regulation of inflammation across evolution.

## Materials and Methods

### Ethics statement

All animals were maintained according to the guidelines of the Canadian Council on Animal Care, and protocols were approved by the University of Alberta Animal Care and Use Committee Committee (ACUC-Biosciences; protocol numbers 760 and 595807). Both goldfish and C57BL/6 mice were terminated via cervical dislocations using approved procedures. All efforts were made to minimize animal stress and to ensure that termination procedures were efficiently performed.

### Animals

Goldfish (*Carassius auratus* L.) 10–15 cm in length were purchased from Mount Parnell (Mercersburg, PA) and maintained in the Aquatic Facility of the Department of Biological Sciences, University of Alberta. The fish were held at 20°C in a flow-through water system on a simulated natural photoperiod. Five-to-nine week old C57BL/6 female mice (*Mus musculus*) were maintained in a P-2-specific pathogen-free facility in the Biosciences Animal Services Centre at the University of Alberta.

### Antibodies

To distinguish murine leukocyte populations, cells were stained with a combination of: CD11b-FITC/Gr1-PE/F4/80-APC or CD11b-PE-Cy7/CD3-FITC/B220-PE/NK1.1-APC. Prior to staining, cells were blocked for 20 min with a solution of 1x PBS^−/−^ with 10% calf serum. All staining was done in a solution of 1x PBS^−/−^ with 2% calf serum for 30 min at room temperature in the dark. Antibodies were added according to the manufacturer's protocols. Goldfish leukocytes were stained with a polyclonal anti-CSF-1R antibody that was produced and validated by the Belosevic lab (University of Alberta) [35]. Cells were stained with 1x PBS^−/−^ with 2% calf serum and 0.05% sodium azide. Following primary stain, goldfish cells were washed and stained with an anti-rabbit-FITC secondary (Jackson ImmunoResearch).

### Goldfish primary kidney macrophage cultures

Primary kidney macrophages (PKM) were generated as previously described [36], [37]. Briefly, total kidney leukocytes were isolated and seeded in 15 mL complete MGFL-15 medium (MGFL-15 supplemented with 100 U/mL penicillin, 100 μg/mL streptomycin, 100 μg/mL gentamicin, 10% newborn calf serum (Gibco) and 5% carp serum), and 5 mL of cell-conditioned medium from previous cultures. Developing cultures were incubated for 7 d at 20°C.

### Preparation of zymosan and apoptotic bodies

Unlabeled zymosan particles (Molecular Probes) were labeled overnight with 75 μg/mL allophycocyanin (APC) (Sigma) with continuous shaking at 4°C in 1x PBS^−/−^ (no magnesium, no calcium). Apoptotic bodies were generated by incubating cells for 24 h in the presence of 10 μg/mL cycloheximide. Treated cells were harvested, washed twice in 1x PBS^−/−^ and stained overnight with 1.5 μg/mL wheat germ agglutinin AlexaFluor 555 (Molecular Probes). Apoptotic bodies were then washed twice in 1x PBS^−/−^. For murine experiments, apoptotic bodies were generated from splenic cells. For goldfish experiments, apoptotic bodies were generated from catfish 3B11 B cells or primary goldfish kidney leukocytes, as indicated. Catfish 3B11 B cells were selected in order to maintain consistency across experiments, as clonal goldfish leukocytes are not available (Figure S1). No difference was found in PKM responses when 3B11 cells were compared with apoptotic bodies derived from primary goldfish kidney leukocytes (Figure S2). Catfish 3B11 cells were a gift from Dr. Melanie Wilson (University of Mississippi Medical Center) and were generated following *in vitro* lipopolysaccharide stimulation of catfish peripheral blood leukocytes with LPS [38], [39].

### Phagocytosis assay

Zymosan-APC and/or apoptotic bodies were added at a ratio of 5∶1 (particle: cell) to 2×10^6^ cells. Mammalian cells were incubated for 2 h at 37°C/5% CO_2_ in complete DMEM medium. Teleost PKM phagocytosis assays were performed in incomplete MGFL-15 media (no serum) at 20°C, as previously described [40]. Following phagocytosis, cells were washed once with 1x PBS^−/−^ and respiratory burst was measured.

### Respiratory burst assay

This assay was performed as previously described [40], [41]. Briefly, following activation, cells were harvested and collected into 5 mL polystyrene round bottom tubes (BD Falcon). Cells were washed twice with 1xPBS^−/−^ then resuspended in 100 µL 1x PBS^−/−^. Dihydrorhodamine (DHR, Molecular Probes) was added to cells at a final concentration of 10 µM and incubated for 5 min to allow cells to take up the DHR. Phorbol 12-myristate 13-acetate (PMA; Sigma) was then added at a final concentration of 100 ng/mL. This concentration of PMA has been shown to trigger ROS production in primed cells [42]. Cells were incubated for a further 30 min to allow oxidation of DHR. All samples were properly staggered with respect to time to accommodate for the transient state of oxidized DHR fluorescence. DHR fluorescence was not quenched by the presence of other fluorochromes, including the wheat germ agglutinin or APC labels on phagocytosed particles (data now shown).

### *In vivo* effects of apoptotic bodies and zymosan

Goldfish and C57BL/6 mice were injected intraperitoneally with 2.5 mg of zymosan (Sigma) in 100 μL of 1x PBS ^−/−^. Animals were sacrificed after 0 h, 8 h, 24 h or 48 h and cells were harvested by peritoneal lavage to determine the time point with maximal cell infiltration based on haemocytometer counts, and respiratory burst. To determine *in vivo* responses to apoptotic bodies, goldfish and C57BL/6 mice were injected with either 5×10^6^ apoptotic bodies (in 100 μL of 1× PBS ^−/−^) and/or 2.5 mg zymosan (Sigma) in 100 μL of 1× PBS ^−/−^ for 24 h. To study the effects of pre-injection of apoptotic bodies, animals were injected with apoptotic bodies 0 h, 2 h, or 4 h before zymosan (zymosan injection defined time 0). Cells were counted using a haemocytometer. Within these time points, changes in cellular numbers were largely associated with cellular infiltration. Sub-populations were defined by expression of specific markers (CD11c, Gr1, F4/80, CD3, B220, NK1.1; mice) or by a combination of size, complexity, morphology and cytochemical staining (goldfish). Respiratory burst was measured by DHR.

### Cytochemical stains

For all cytochemical stains, 1×10^5^ cells were spun onto glass slides at 55×*g* using a cytocentrifuge (Shandon Instruments) and fixation and staining for modified Wright-Giemsa stain and Sudan Black were carried out according to the manufacturers protocols. Briefly, for modified Wright's staining (Hema3, Fisher Scientific), cells were fixed in 70% methanol for 1 min, stained in hematoxylin for 1 min and counterstained in eosin for 40 sec. For Sudan Black staining (Sigma), cells were fixed for 1 min in an acetone-glutaraldehyde solution. Cells were then stained for 5 min with Sudan Black followed by a 5 min counterstain with hematoxylin. Photomicrographs were generated using a DM1000 microscope (Leica) using a bright field 100× objective (1000× magnification). Images were acquired using QCapture software.

### Isolation of neutrophil and monocyte/macrophage populations to determine effects of apoptotic bodies on isolated populations

Peritoneal cells were lavaged from goldfish or C57BL/6 mice injected with 2.5 mg of zymosan 24 h earlier. Goldfish lavages were run over a 51% Percoll gradient. Murine lavages were run over a Ficoll (Histo-Paque) gradient. Following centrifugation, buffy coats containing mononuclear cells were collected into a separate tube. These cells and the neutrophil pellets were washed with once with serum-free media. Cells were then resuspended in complete media and plated in 6-well plates with 1×10^6^ cells in 2 mL in each condition (responding cells). Transwells (0.4 μm pore size, Corning) were added to the specified wells, 1 mL of media was added into each transwell, followed by activator cells and/or apoptotic bodies. Cells were then incubated for 2 h (at 20°C for goldfish cells; 37°C/5% CO_2_ for murine cells). Five μg/mL zymosan was added to all murine cells during the incubation to maintain activation achieved *in vivo*. Following incubation, cells were harvested and ROS production was assayed with DHR. Isolated neutrophil and mononuclear cells were also used in the phagocytosis assay described above. Since lymphocytes could not be efficiently removed from mononuclear cells preparations without affecting cellular responses, these cells were removed from the analysis through gating strategies. As such, analyzed populations are referred to as ‘neutrophils’ and ‘monocytes/macrophages’.

### Analysis

ImageStream data was analyzed using IDEAS software (Amnis), as previously described [40]. Flow cytometry data was acquired on a FACSCanto II flow cytometer (BD Biosciences) and data was analyzed using FACSDiva or FCS Express v3 software. Statistics were performed by Students' T-test using Prism 4 software (GraphPad Prism).

## Results

### Teleost phagocytes reduce pro-inflammatory responses following apoptotic body internalization

**Figure 1:**
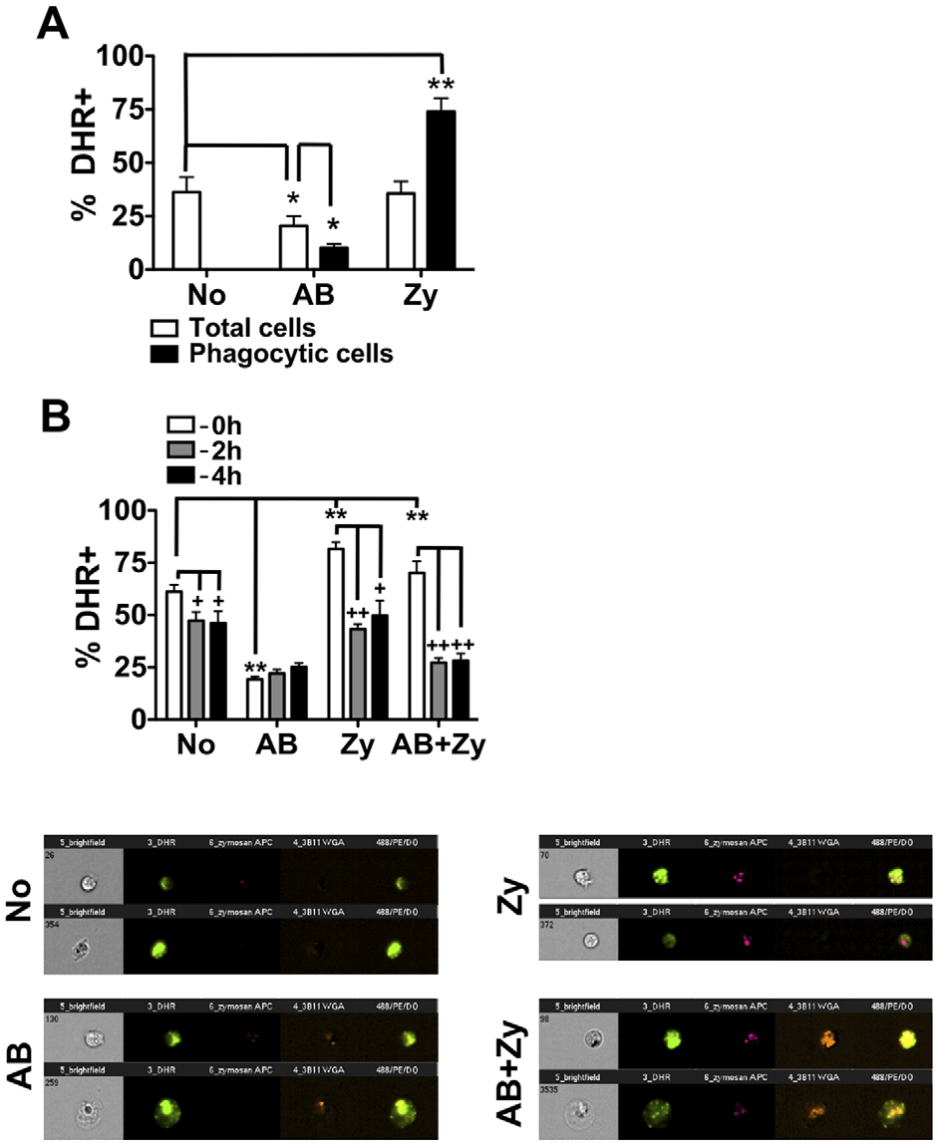
Apoptotic bodies significantly reduce inflammatory respiratory burst responses of goldfish primary macrophages. (A) Goldfish PKM were separately incubated with apoptotic bodies or zymosan (5∶1, particle: cell ratio) for 2 h. Cells were then harvested and respiratory burst was assayed using DHR. n = 3. (B) Goldfish PKM were cultured in the presence of both apoptotic bodies and zymosan for 2 h (5∶1 ratio for both). Respiratory burst was then analyzed in cells based on phagocytic capacity as follows: non-phagocytic cells, cells containing only apoptotic bodies, cells containing only zymosan or cells containing both apoptotic bodies and zymosan (white bars). Representative images are shown for cells in each of these groups. To investigate the effects of pre-incubation with apoptotic bodies, apoptotic bodies were added to PKM simultaneously to zymosan (‘−0 h’), 2 h before zymosan (‘−2 h’) or 4 h before zymosan (‘−4 h’). n = 4. For all, * p<0.05; ** p<0.01; For (c), + p<0.05 and ++ p<0.01 compared to (‘−0 h’). No- no internalized particle; AB- apoptotic body; Zy- zymosan.

As a first step in the characterization of divergent pro-inflammatory and homeostatic responses in lower vertebrate phagocytes, we examined the impact of zymosan and apoptotic body internalization in a well-characterized phagocyte model of teleosts that uses primary macrophages (PKM) derived from the hematopoietic compartment of goldfish [36], [37]. Cellular respiratory burst was used as a marker for the induction of macrophage antimicrobial responses. This also provided important insights into NADPH activity, which is known to have key roles in both the induction and resolution of inflammation. As expected, internalization of zymosan induced a strong respiratory burst among phagocytes (Figure 1A). Conversely, phagocytosis of apoptotic bodies resulted in a significant decrease in the production of reactive oxygen species (ROS). Interestingly, zymosan selectively increased the respiratory burst in phagocytes internalizing zymosan, while the presence of apoptotic bodies decreased the respiratory burst responses in both phagocytic and non-phagocytic cells (Figure 1A). Internalization of apoptotic bodies may induce soluble mediators in fish macrophages to globally reduce activation.

Within an inflammatory site, macrophages are exposed to both pro-inflammatory stimuli and dying cells. As such, we measured the effects on macrophage activation following combined stimulation with both apoptotic bodies and zymosan. Individual goldfish PKM respiratory burst responses were analyzed by imaging flow cytometry based on cells that had no particles internalized, cells that had only apoptotic bodies internalized, cells that had only zymosan internalized, or cells that had internalized both apoptotic bodies and zymosan (Figure 1B). We observed the same trends as when cells were treated with a single stimulus despite the presence of four distinct phagocyte groups within these mixed populations – PKM that ingested apoptotic bodies exhibited a significant decrease in respiratory burst while PKM with internalized zymosan had significantly increased respiratory burst (Figure 1B; refer to 0 h group). Together with results from Figure 1A, this data indicates that the divergence of responses to pro-inflammatory and homeostatic signals at the level of the individual phagocyte is already present in teleost fish. Further, our results indicate that regulation of pro- and anti-inflammatory phagocyte responses displays an important intrinsic level of regulation.

**Figure 2:**
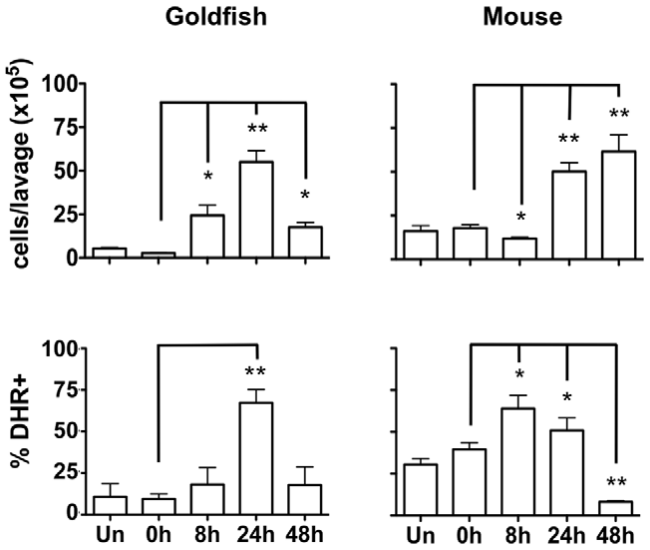
*In vivo* administration of zymosan induces a marked infiltration of leukocytes that is linked to high levels of respiratory burst. Goldfish (left) and C57BL/6 mice (right) were injected intraperitoneally with 2.5 mg of zymosan. Cells were harvested by peritoneal lavage at 0 h (saline alone), 8, 24 and 48 h and counted (top row). Injection of zymosan resulted in a marked increase in cell numbers isolated from the peritoneum that peaked at 48 h for mice and 24 h for goldfish. Respiratory burst in isolated cells at these time points was determined with DHR (bottom row). n = 4; * p<0.05; ** p<0.01.

Pre-incubation with apoptotic bodies for 2 h or 4 h prior to addition of zymosan resulted in reduced respiratory burst responses in those cells that were non-phagocytic, those that had only internalized zymosan or those that had internalized both zymosan and apoptotic bodies (Figure 1B, −2 h and −4 h groups), again suggesting the involvement of soluble factors for amelioration of pro-inflammatory responses. For the group that had only internalized apoptotic bodies, pre-incubation did not lead to an added decrease in ROS production, suggesting that maximal down-regulation of respiratory burst responses was achieved by 2 h. Together, these data show that internalization of apoptotic bodies leads to discrete cellular events that result in reduced macrophage activation in the presence or absence of stimulatory zymosan particles. The former indicates that this control mechanism(s) is sufficient to over-ride stimulatory signals that would otherwise lead to pro-inflammatory responses.

### Zymosan differentially induces cellular infiltration in mice and teleost fish

**Figure 3:**
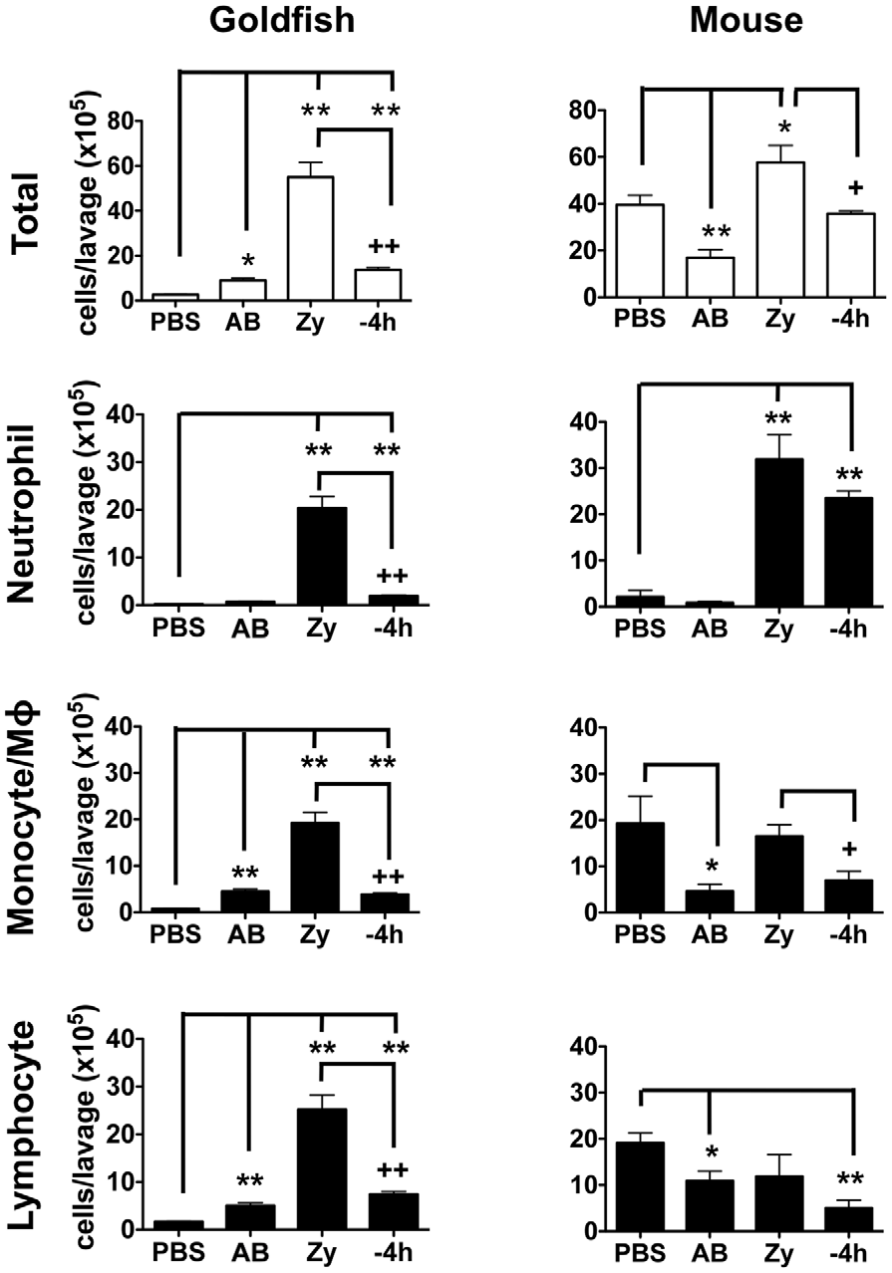
Pro-inflammatory (zymosan) and homeostatic (apoptotic bodies) stimuli differentially impact leukocyte infiltration profiles in goldfish and mice. Goldfish and C57BL/6 mice were injected intraperitoneally with saline, apoptotic bodies (5×10^6^) or zymosan (2.5 mg). Apoptotic bodies were also pre-injected 4 h before zymosan injections. Goldfish leukocyte populations were defined by imaging flow cytometry (area, internal complexity, and morphology) and staining patterns with Sudan Black and an anti-CSF-1R antibody (Figure S3). Murine cells were defined based on surface markers for neutrophils (F4/80^−^/Gr1^+^/CD11b^+^), monocytes (F4/80^lo^/Gr1^+/−^/CD11b^+^), macrophages (F4/80^hi^/Gr1^+/−^/CD11b^+^) and lymphocytes (F4/80^−^/Gr1^−^; CD3, B220, NK1.1; Figure S4A). n = 4; * p<0.05 and ** p<0.01 compared to control; + p<0.05 and ++ p<0.01 compared to zymosan. No- no internalized particle; AB- apoptotic body; Zy- zymosan.

Cellular infiltration and induction of antimicrobial defenses are two of the hallmarks of inflammation at an infection site [4]. Using a commonly used zymosan-peritonitis model, we determined that 24 h following injection was the best time point for comparison of cellular infiltration and ROS responses between goldfish and mice; at this time point both had reached significant increases in the number of infiltrating leukocytes (Figure 2). However, examination of leukocyte exudates following intraperitoneal challenge with zymosan showed marked differences between mice and goldfish. Murine cellular infiltrates were defined based on surface expression of Gr1^+^/CD11b^+^/F4/80^−^ for granulocytes, Gr1^+/−^/CD11b^+^/F4/80^mid/hi^ for monocytes/macrophages, and CD3, B220, and NK1.1 expression for lymphocytes (Figure S3). In the absence of the range of surface markers available for mice, goldfish leukocytes were first characterized based on imaging flow cytometry parameters. Because neutrophils displayed similar morphology to monocytes [43], cytochemical staining and surface receptor expression further defined the leukocyte subsets isolated from the goldfish peritoneum (Figure S4). Injected goldfish displayed dramatic increases in the total numbers of infiltrating leukocytes, comprised of both myeloid and lymphoid cells (an overall 20-fold relative to saline controls; Figure 3). Increases were noted in neutrophils, monocytes/macrophages, and lymphocytes (20-fold, 25-fold, and 15-fold increases relative to saline controls, respectively). Results based on Sudan Black cytochemical staining were further supported by a significant parallel increase in the expression of the granulocyte colony stimulating factor-receptor in peritoneal infiltrates based on quantitative PCR 24 h following zymosan administration (data not shown).

In contrast to goldfish, mice displayed a more moderate increase in the total number of infiltrating leukocytes compared to saline controls (1.5-fold increase; Figure 3), with the increase limited to cells in the neutrophil pool (Gr1^+^/CD11b^+^/F4/80^−^). No significant changes were detected in the infiltration of monocyte/macrophage (Gr1^+/−^/CD11b^+^/F4/80^mid/hi^) or lymphocyte (based on CD3, B220, and NK1.1 expression) subsets. Our data suggests that inflammatory responses in the murine peritoneum may depend to a greater extent on resident leukocytes and/or targeted infiltration by specific leukocyte subsets. Importantly, we also found significant differences in the kinetics of these leukocyte infiltration responses. Faster kinetics were observed in goldfish (rapid induction by 8 h) compared to a delayed but sustained onset of leukocyte infiltration in mice from 24 to 48 h (Figure 2).

### Apoptotic bodies differentially control cellular infiltration in mice and teleost fish

*In vivo* peritoneal administration of apoptotic bodies also led to significant differences for the modulation of leukocyte infiltration in our lower and higher vertebrate models. In goldfish, addition of apoptotic bodies led to a 3.2-fold increase in total number of infiltrating leukocytes compared to saline controls (goldfish total leukocyte panel; Figure 3). This was driven by increases in monocyte/macrophage and lymphocyte populations. No change was detected in the neutrophil pool. In sharp contrast, administration of apoptotic bodies to mice induced a significant decrease in leukocyte infiltration compared to saline controls, driven by a reduced number in monocyte/macrophage and lymphocyte populations (Figure 3). No change was detected in the neutrophil pool. Thus, upon immune recognition of apoptotic bodies, mice and goldfish differentially modulate the recruitment of individual leukocyte populations.

The presence of apoptotic bodies also showed differential effects on cellular infiltration driven by zymosan in mice and goldfish. Overall, administration of apoptotic bodies returned levels of leukocyte infiltration to those of saline controls in both goldfish and mice (Total: −4 h versus PBS groups; Figure 3). However, significant differences were detected, particularly when focused on the neutrophil subset. Goldfish showed efficient down-modulation of neutrophil infiltration when apoptotic bodies were administered to a zymosan-induced inflammatory site (neutrophil group; Figure 3). In contrast, parallel experiments in mice showed maintenance of neutrophil numbers (Figure 3). This may point to an increased role for infiltrating neutrophils in the control of inflammation in higher vertebrates, which goes beyond the initial induction and maintenance of early pro-inflammatory phases of the antimicrobial response. This is consistent with the resistance of transmigrated inflammatory neutrophils to pro-survival/anti-apoptotic signals, potentially to allow the mammalian host to better take advantage of apoptosis as an effective mechanism for the control of inflammation [44].

### Apoptotic bodies reduce teleost pro-inflammatory responses in vivo in a time dependent manner

**Figure 4:**
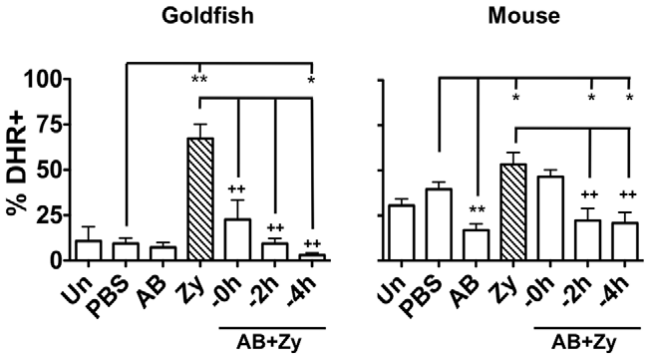
*In vivo* administration of apoptotic bodies leads to a more dramatic reduction of pro-inflammatory respiratory burst responses in teleost fish compared to mice. Goldfish and C57BL/6 mice were injected intraperitoneally with saline, apoptotic bodies (5×10^6^) or zymosan (2.5 mg) and incubated for 24 h. Apoptotic bodies were also pre-injected 0, 2, or 4 h before zymosan injections to assess the contributions of kinetics to these responses. Cells from injected animals were harvested by peritoneal lavage and respiratory burst was assayed with DHR. n = 4; * p<0.05 and ** p<0.01 compared to PBS (saline) control; + p<0.05 and ++ p<0.01 compared to zymosan. No- no internalized particle; A.B.− apoptotic body; Zy- zymosan.

**Figure 5:**
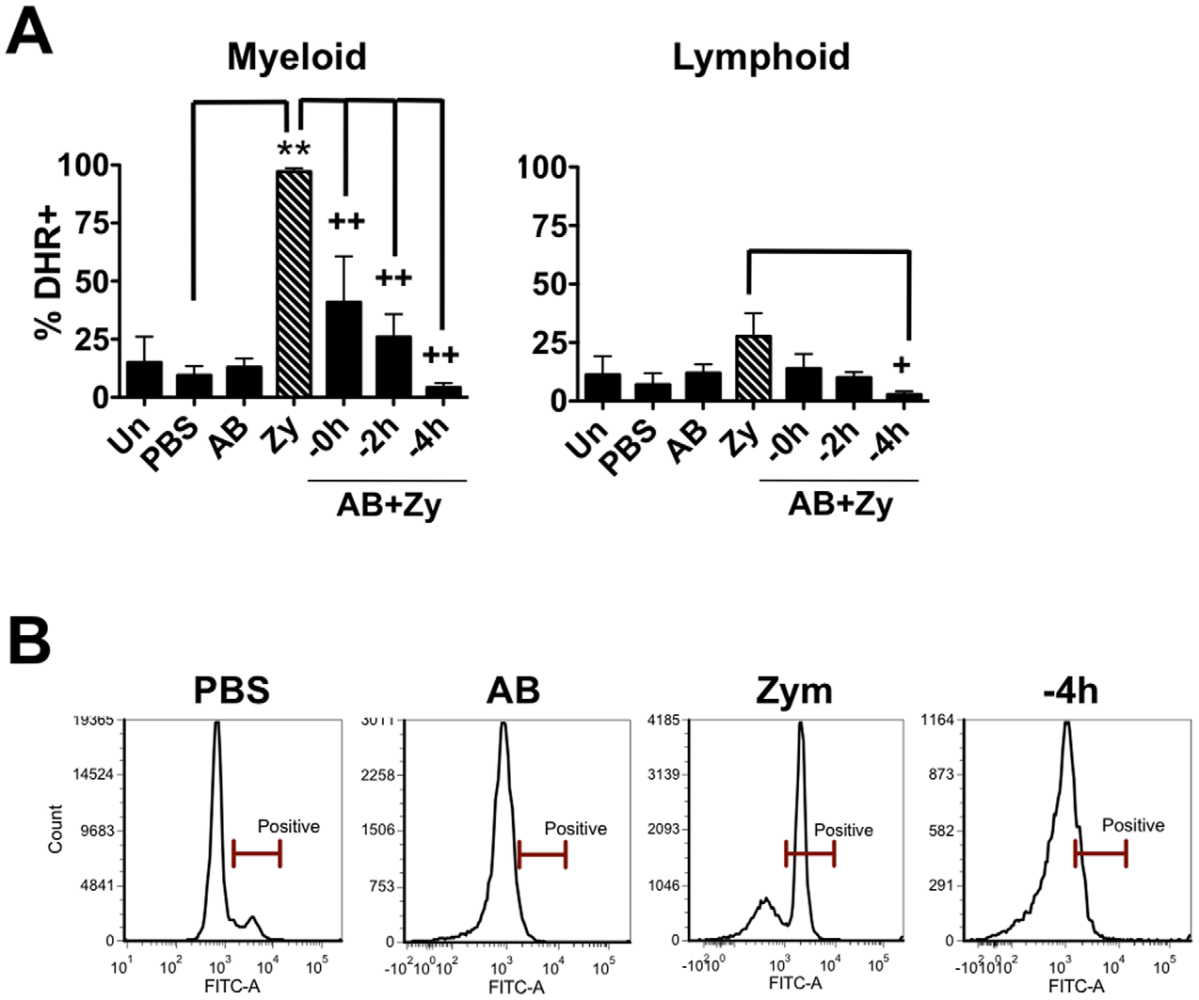
Goldfish myeloid cell respiratory burst responses are most affected by the presence of apoptotic bodies. (A) Goldfish were injected intraperitoneally with saline, apoptotic bodies (5×10^6^) or zymosan (2.5 mg). Apoptotic bodies were also pre-injected 0, 2, or 4 h before zymosan injections. Cells from injected goldfish were harvested by peritoneal lavage and respiratory burst was assayed with DHR in peritoneal cell subpopulations based on forward scatter and side scatter profiles. n = 4; * p<0.05 and ** p<0.01 compared to control; + p<0.05 and ++ p<0.01 compared to zymosan. No- no internalized particle; A.B.- apoptotic body; Zy- zymosan. (B) Representative histograms show a single peak in DHR responses.

Consistent with *in vitro* measurements, injection of zymosan led to significant increases in respiratory burst in goldfish peritoneal cells (Figure 4). Co-injection of apoptotic bodies significantly decreased the respiratory burst relative to the ‘zymosan only’ group. The respiratory burst response decreased in a time dependent manner, with respiratory burst being almost undetectable at the −4 h time point despite the presence of zymosan. As expected, respiratory burst responses predominantly occurred in the myeloid, and not lymphoid population (Figure 5). Mean fluorescence intensity of myeloid cell DHR fluorescence shows a similar trend (Figure S5, top). Interestingly, the decrease in respiratory burst responses was more pronounced *in vivo* than *in vitro* (Figure 1B vs. Figure 4), suggesting that teleost cells at an inflammatory site other than macrophages contribute to the control of pro-inflammatory responses.

### Apoptotic bodies reduce pro-inflammatory responses in mice to a lesser extent than in goldfish

**Figure 6:**
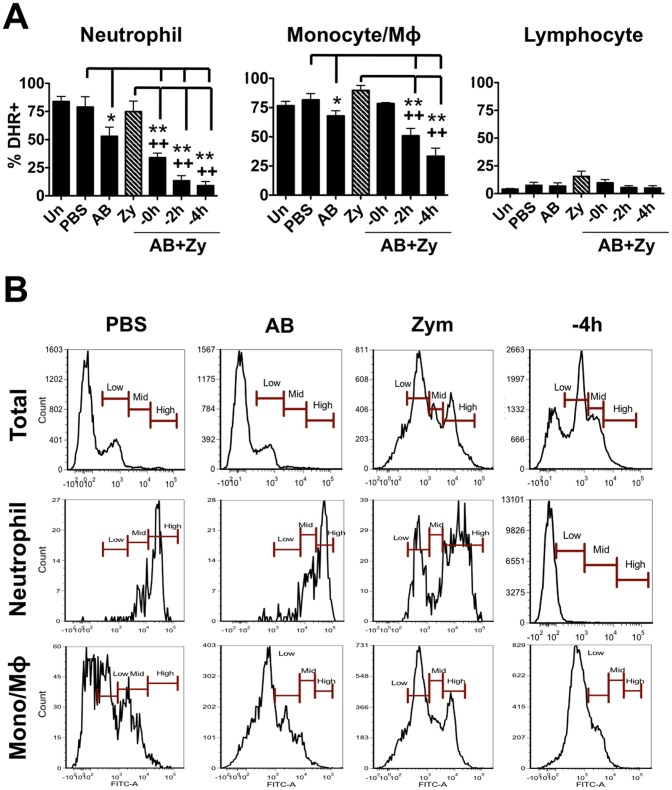
Murine neutrophil respiratory burst antimicrobial responses are most greatly affected by the presence of apoptotic bodies. (A) C57BL/6 mice were injected intraperitoneally with saline, apoptotic bodies (5×10^6^) or zymosan (2.5 mg). Apoptotic bodies were also pre-injected 0, 2, or 4 h before zymosan injections. Cells from injected mice were harvested by peritoneal lavage and respiratory burst was assayed with DHR in peritoneal cell subpopulations based on forward scatter and side scatter profiles (Figure S4B). n = 4; * p<0.05 and ** p<0.01 compared to control; + p<0.05 and ++ p<0.01 compared to zymosan. No- no internalized particle; A.B.- apoptotic body; Zy- zymosan. (B) Histograms show representative DHR responses for total leukocytes, neutrophils and monocytes/macrophages. We found that the high responders were predominantly neutrophils (>90%). Pre-incubation with apoptotic bodies resulted in a preferential switch in the neutrophil population from high responders to mid/low responders.

Although *in vitro* and *in vivo* studies of murine immune responses to either zymosan [45], [46] or apoptotic bodies [8], [10] have been examined independently, the combined effects have not been thoroughly investigated. To this end, we injected C57BL/6 mice intraperitoneally with zymosan and/or apoptotic bodies. Similar to goldfish, zymosan injections of mice resulted in respiratory burst activity in peritoneal cells (Figure 4); however, basal responses were higher and zymosan-induced responses were not as pronounced as those in goldfish (ie. relative increase in ROS production in response to zymosan was much less pronounced in mice). Mice injected with apoptotic bodies also exhibited significantly reduced respiratory burst response compared to those in the zymosan-injected group (Figure 4). Again, the relative decrease was less pronounced than that observed in goldfish. Interestingly, murine neutrophil populations demonstrated the greatest decrease in ROS production, while the monocyte/macrophage subset showed mild reductions in the respiratory burst (Figure 6A). This decrease was further associated with a shift in neutrophil ROS responses from ‘high’ levels to ‘low’/‘mid’ levels, especially in neutrophils from mice injected with apoptotic bodies 4 h prior to zymosan (Figure 6B). A similar shift was noted in the monocyte/macrophage population, although it was not as prominent as that of neutrophils. Mean fluorescence intensity of neutrophil and monocyte/macrophage DHR fluorescence shows a similar trend to these results (Figure S5, bottom).

### Murine, but not teleost, neutrophils phagocytose apoptotic bodies, which actively reduce pro-inflammatory ROS production in a contact dependent manner

**Figure 7:**
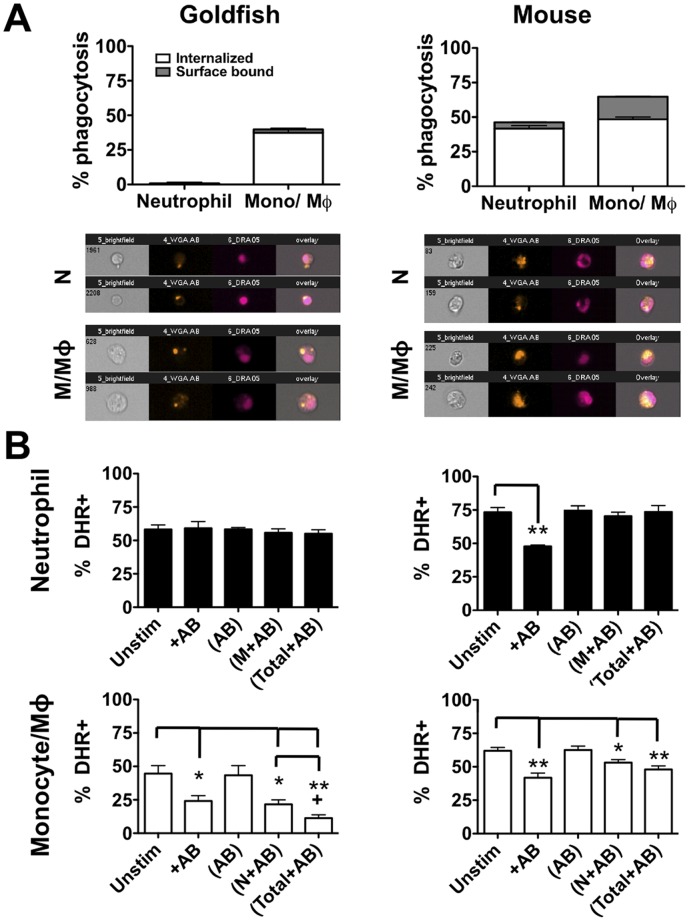
Apoptotic bodies downregulate murine neutrophil ROS production in a contact dependent manner. Goldfish (left) and C57BL/6 mice (right) were injected intraperitoneally with zymosan (2.5 mg). Activated peritoneal cells from were harvested by peritoneal lavage and subpopulations were isolated by density centrifugation. (A) Separated neutrophil or mononuclear populations were incubated with labeled apoptotic bodies for 2 h and internalization was analyzed. n = 4; * p<0.05 and ** p<0.01; Mφ/M  =  macrophage/monocyte. (B) Isolated populations were cultured for 2 h in the presence of the indicated stimuli. Conditions denoted within brackets were contained within a 4 μm transwell. After 2 h, responder cells outside the transwells were harvested and respiratory burst was assayed using DHR.

Mammalian macrophages are well known to internalize apoptotic bodies and subsequently down-regulate their pro-inflammatory responses [5]–[7]. Similarly, previous reports indicate that activated *ex vivo* human neutrophils possess the capacity to phagocytose apoptotic cells, which results in a decrease in pro-inflammatory neutrophil functions, including production of ROS [47]. This establishes macrophages and neutrophils as active contributors to homeostatic responses at a mammalian inflammatory site. However, classical neutrophils represent recent additions to the immune cell repertoire of metazoans and, therefore, may have only recently acquired the capacity to contribute to both pro-inflammatory and homeostatic responses in higher vertebrates. Therefore, we wished to compare the relative capacity of murine and teleost neutrophils to internalize apoptotic bodies, and to determine if this subsequently decreased ROS production through either contact dependent or soluble factor-mediated mechanisms. To this end, goldfish and C57BL/6 mice were injected intraperitoneally with zymosan and cells were harvested by peritoneal lavages 24 h later. Using density gradient centrifugation, isolated cells were separated into granulocyte and mononuclear fractions in a manner that has been shown to result in a high level of purity in the isolated populations [43]. To determine the capacity of these leukocytes to internalize apoptotic bodies, cells were incubated with labeled species-specific apoptotic bodies for 2 h and phagocytosis was analyzed. Consistent with murine results, goldfish monocytes/macrophages were efficient at internalizing apoptotic bodies (Figure 7A). There was also limited surface binding capacity in both goldfish myeloid populations. In sharp contrast, goldfish neutrophils displayed a very limited capacity for their uptake (<0.6% phagocytosis) whereas murine neutrophils displayed efficient internalization of apoptotic bodies equivalent to that of murine monocytes/macrophages.

To measure the effects of apoptotic bodies on activated *ex vivo* neutrophils and monocyte/macrophage populations, cells were stimulated *in vivo* and isolated as described above. Cells were then cultured for 2 h in the presence of apoptotic bodies. To determine if the effect was contact dependent or mediated through soluble factors, apoptotic bodies were also added to 0.4 μm transwells alone or in combination with various cell populations (Figure 7B; bracketed groups). In the transwell conditions, only responding cells were present outside of the transwell (ie. no other factors were added to these cells). In goldfish, neutrophil ROS production was unchanged in any of the experimental conditions, indicating that neither contact dependent factors nor soluble mediators were present and affected neutrophil responses (Figure 7B, left panel). In contrast, direct contact with apoptotic bodies significantly reduced monocyte/macrophage ROS production. This suggests that the reduction in ROS production identified in the myeloid population of Figure 5 was associated with a selective decrease in monocyte/macrophage and not neutrophil ROS production. Interestingly, in spite of the limited ability of neutrophil to phagocytose apoptotic bodies, activated goldfish neutrophils cultured in the presence of apoptotic bodies produced soluble factor(s) that significantly decreased monocyte/macrophage ROS responses (bracketed N+AB group in monocyte/macrophage analysis; Figure 7B). This decrease is further enhanced when the total peritoneal isolate was used, suggesting an additive role of soluble factors from both neutrophils and mononuclear cells (bracketed Total+AB group in monocyte/macrophage analysis; Figure 7B).

In sharp contrast, isolated murine neutrophils significantly downregulated their ROS responses in the presence of apoptotic bodies (Figure 7B, right panel), similar to the responses observed following *in vivo* injection of apoptotic bodies. The downregulation of ROS was contact-dependent, as no changes in ROS were observed when apoptotic bodies alone, monocyte/macrophages plus apoptotic bodies, or total leukocytes plus apoptotic bodies were added to the experimental transwell. This suggests that the decreases in neutrophil ROS measured *in vivo* were due to direct contact between apoptotic bodies and neutrophils within the peritoneum and not soluble factors produced by other leukocytes at the site.

## Discussion

Phagocytosis is a well-conserved innate defense mechanism that has served as a robust platform for incorporation of novel layers of immunological control [11], [20], [22], [23]. For inflammatory processes of higher vertebrates, phagocytosis represents a central node for the induction and resolution of inflammation. In this manuscript, we used murine and teleost fish *in vivo* and *in vitro* models to assess the evolutionary conservation of this inflammation control axis at an inflammatory site.

Although we found some conservation of phagocyte functional responses, we also found significant differences in the level and control of these responses to pro-inflammatory and homeostatic internalization signals between goldfish and mice. Fish mounted very robust, quick responses to zymosan that led to the recruitment of several leukocyte populations. Addition of apoptotic bodies resulted in a marked suppression of these responses. In contrast, mice displayed more subtle responses to both zymosan and apoptotic bodies that targeted cellular recruitment and appeared to rely more heavily on resident tissue subsets.

Two important characteristics of an inflammatory site are cellular infiltration and induction of antimicrobial defenses [4]. Goldfish exhibited significantly higher cellular infiltration than mice following intraperitoneal challenge with zymosan (20-fold increase vs 1.5 fold increase). In goldfish, this infiltrate was composed of both lymphoid and myeloid cells, while murine leukocyte infiltration was dominated by neutrophils. Furthermore, apoptotic bodies inhibited infiltration of both myeloid and lymphoid cells in goldfish. This is in sharp contrast to mice, where the infiltrating macrophage and lymphocyte populations modestly decreased while neutrophil infiltration was maintained in the presence of apoptotic body stimuli.

Murine respiratory burst responses also displayed added cell specificity compared to our teleost model. In mice, apoptotic bodies preferentially caused a decrease in neutrophil respiratory burst, whereas only a limited effect was evident in macrophage responses. Murine respiratory burst responses also displayed faster kinetics preceding the wave of leukocyte infiltration, suggesting a significant contribution from resident peritoneal leukocytes. Our results are consistent with an evolutionary shift towards leukocyte specialization in mammals. This shift appears to favor targeted induction of leukocyte infiltration (F4/80^−^/Gr1^+^/CD11b^+^ neutrophils in this model), and is increasingly reliant on local peritoneal leukocyte populations for early activation of innate antimicrobial effector mechanisms. This may be associated with evolving roles for specific leukocyte populations. For example, teleost circulating lymphocyte pools contribute directly to innate phagocytic responses [48]–[50], whereas mammalian lymphocytes display increased specialization towards homeostatic tissue repair mechanisms [51]–[54] and adaptive mechanisms of immunity [55]–[57].

Our results also point to changing roles for neutrophils across evolution. In both goldfish and mice, neutrophils are among the first leukocytes recruited to an inflammatory site [58] and their loss from an inflammatory site is often considered a key histological feature that signals the start to the resolution of inflammation [59]. The evolutionary origins of neutrophils may lie in their roles as novel contributors for the induction of potent antimicrobial defenses. However, recent studies highlight another role for mammalian neutrophils as contributors to homeostatic responses at an inflammatory site. Within hours after entering an inflammatory site, infiltrating neutrophils actively produce protective molecules, such as lipoxins from arachidonic acid, which promote resolution of an inflammatory response (coined ‘lipid mediator class- switching’) [59]. Our results add to this paradigm and indicate that mammalian neutrophils display an increased capacity to participate in the down-regulation of inflammation though contact dependent interactions with apoptotic bodies. This contact and subsequent internalization of apoptotic bodies significantly reduces neutrophilic as well as macrophage ROS production through a putative soluble factor-mediated mechanism. The identity of this soluble factor(s) is currently unknown but lipoxins have been shown to attenuate ROS production in a number of cells including microglia [60], endothelial cells [61], and aggregated peripheral blood leukocytes [62]. Further, while the ability of neutrophils to internalize apoptotic bodies appears to have evolved recently, teleost neutrophils are able to produce soluble factor(s) that downregulate macrophage ROS production. As such, future studies should assess the potential differences between mammals and teleosts in the production of pro-resolution molecules such as lipoxins, resolvins, and protectins, as well as the role(s) that these molecules may have in the resolution of inflammation through neutrophil/apoptotic body contact-dependent and independent mechanisms.

The balance between pathogen and homeostatic phagocytic responses is critical for the effective induction of pathogen clearance mechanisms that are efficiently controlled to prevent excessive tissue damage [63]. Loss of this balance contributes to the development and exacerbation of autoimmune diseases that include systemic lupus erythematosus [64], adult respiratory distress syndrome and rheumatoid arthritis [65], and can also lead to incomplete pathogen clearance [4]. The specific importance of homeostatic phagocytic responses is further highlighted by several recent studies. For example, injection of apoptotic bodies has been shown to prevent the development of experimental immune complex-mediated inflammatory arthritis [66], accelerate the resolution of acute inflammation [8], expand a pool of TGF-β tolerogenic T cells [67], non-specifically facilitate allogenic bone marrow engraftment [68], and interfere with graft rejection and the development of graft-versus-host disease [69]. These effects appear to be linked to the capacity of exogenously administered apoptotic bodies to promote an immunosuppressive environment, activate immunomodulatory cells, and decrease inflammatory immune cell infiltration [8]. We have shown here for the first time that the presence of apoptotic bodies results in greatly diminished zymosan-induced ROS production. This provides added insights into the mechanisms by which apoptotic bodies contribute to the control of inflammatory processes and may also highlight unique therapeutic opportunities for treatment of those disease states that possess a strong ROS component.

Together, our results demonstrate that the distinct polarization of immune cells in response to inflammatory or homeostatic stimuli are evolutionarily intact in bony fish. However, differences in the rate of leukocyte migration to the inflammatory site, increased selectivity for the leukocyte subsets recruitment to this site, increased participation of resident leukocyte pools, and changes to the kinetics and strength of the antimicrobial response highlight the significant honing of that has developed for the control of inflammation across evolution.

## Supporting Information

Figure S1 **Cycloheximide primarily induces apoptosis in treated cells and can be effectively removed from apoptotic body preparations.** (A) Representative experiments show apoptotic bodies generated for treatment of goldfish phagocytes. 3B11 catfish B cells were cultured for 24 h in the presence of 10 μg/mL cycloheximide. Cells were subsequently harvested and stained with Annexin V/propidium iodide to determine cell viability. Cycloheximide treatment primarily induced apoptotic cell death for effective generation of apoptotic bodies, which were then labeled with wheat germ agglutinin-Alexa Fluor 555 overnight. (B) To ensure that apoptotic body preparations did not negatively impact phagocyte viability, apoptotic body preparations were washed three times in 1x PBS^−/−^ to remove remaining cycloheximide and added to goldfish PKM for the times indicated. At these time points, PKM cells were harvested and stained with Annexin V/propidium iodide to assess viability status of these phagocytes. Apoptotic bodies did not induce cell death in PKM cultures.
(TIF)

Figure S2 **Two distinct sources of apoptotic bodies repress goldfish macrophage respiratory burst to equivalent levels.** PKM cultures were incubated with apoptotic bodies derived from 3B11 B cells or goldfish kidney leukocytes. Cells were incubated for 2 h (5∶1, particle: cell ratio) and respiratory burst was analyzed by DHR. There was no significant difference in PKM responses to 3B11-derived or kidney-derived apoptotic bodies.
(TIF)

Figure S3 **Gating strategy for cell subpopulations isolated from murine peritoneum.** (A) Peritoneal cells from C57BL/6 mice were stained with combination of CD11b-FITC/Gr1-PE/F4/80-APC or CD11b-PE-Cy7/CD3-FITC/B220-PE/NK1.1-APC to determine the infiltration of granulocytes (F4/80^−^/Gr1^+^/CD11b^+^), macrophages (F4/80^hi^/Gr1^+/−^/CD11b^+^), monocytes (F4/80^lo^/Gr1^+/−^/CD11b^+^) and lymphocytes (F4/80^−^/Gr1^−^). Lymphocyte populations were confirmed to contain T cells (CD11b^−^/CD3^+^), B cells (CD11b^+/−^/B220^+^) and NK cells (CD11b^−^/NK1.1^+^). (B) Murine peritoneal cells were stained with DHR and analyzed using a FACSCanto II flow cytometer. Cell populations were determined based on forward (FSC-A) and side scatted (SSC-A) characteristics.
(TIF)

Figure S4 **Characterization of goldfish peritoneal myeloid cells.** (A) Total peritoneal exudates were analyzed by imaging flow cytometry and four distinct cellular subsets characterized based on area, internal complexity, and morphology. Unlike macrophage and lymphocyte subsets, monocytes and neutrophils could not be subdivided into two distinct populations solely based on these parameters. Modified Wright's stain confirmed the presence of cells with classical lymphocyte, neutrophil, and monocyte/macrophage morphology. (B) To better differentiate between myeloid populations within the goldfish peritoneal exudate, cells were analyzed based on surface CSF-1R expression and Sudan Black cytochemical staining, which denote monocyte/macrophages and neutrophils, respectively. Representative cells stained with anti-CSF-1R antibodies or Sudan Black are shown. Goldfish were injected intraperitoneally with saline, apoptotic bodies (AB; 5×10^6^) or zymosan (Zy; 2.5 mg) and incubated for 24 h. Apoptotic bodies were also pre-injected 4 h (−4 h) before zymosan injections to assess the contributions of kinetics to these responses. For flow cytometry, n = 2; for cytochemical stains, n = 4. * p<0.05 and ** p<0.01 compared to control; ++p<0.01 compared to zymosan.
(TIF)

Figure S5 **Mean fluorescence intensity of teleost and murine phagocytes.** Goldfish and mice were injected intraperitoneally with saline, species-specific apoptotic bodies (5×10^6^) or zymosan (2.5 mg). Apoptotic bodies were also pre-injected 4 h before zymosan injections. Cells from injected animals were harvested by peritoneal lavage and respiratory burst was assayed with DHR in peritoneal cell subpopulations based on forward scatter and side scatter profiles. The mean fluorescence intensity was calculated based on the mean DHR fluorescence in the entire population. For goldfish, myeloid cells are shown. For mice, phagocyte populations were further split into neutrophils and monocyte/macrophages.
(TIF)
