# Supplementary figures and images for: Fish and Mammalian Phagocytes Differentially Regulate Pro-Inflammatory and Homeostatic Responses In Vivo
Source: PLoS One. 2012 Oct 23;7(10):e47070. doi: 10.1371/journal.pone.0047070 (PMC3479104; doi:10.1371/journal.pone.0047070)

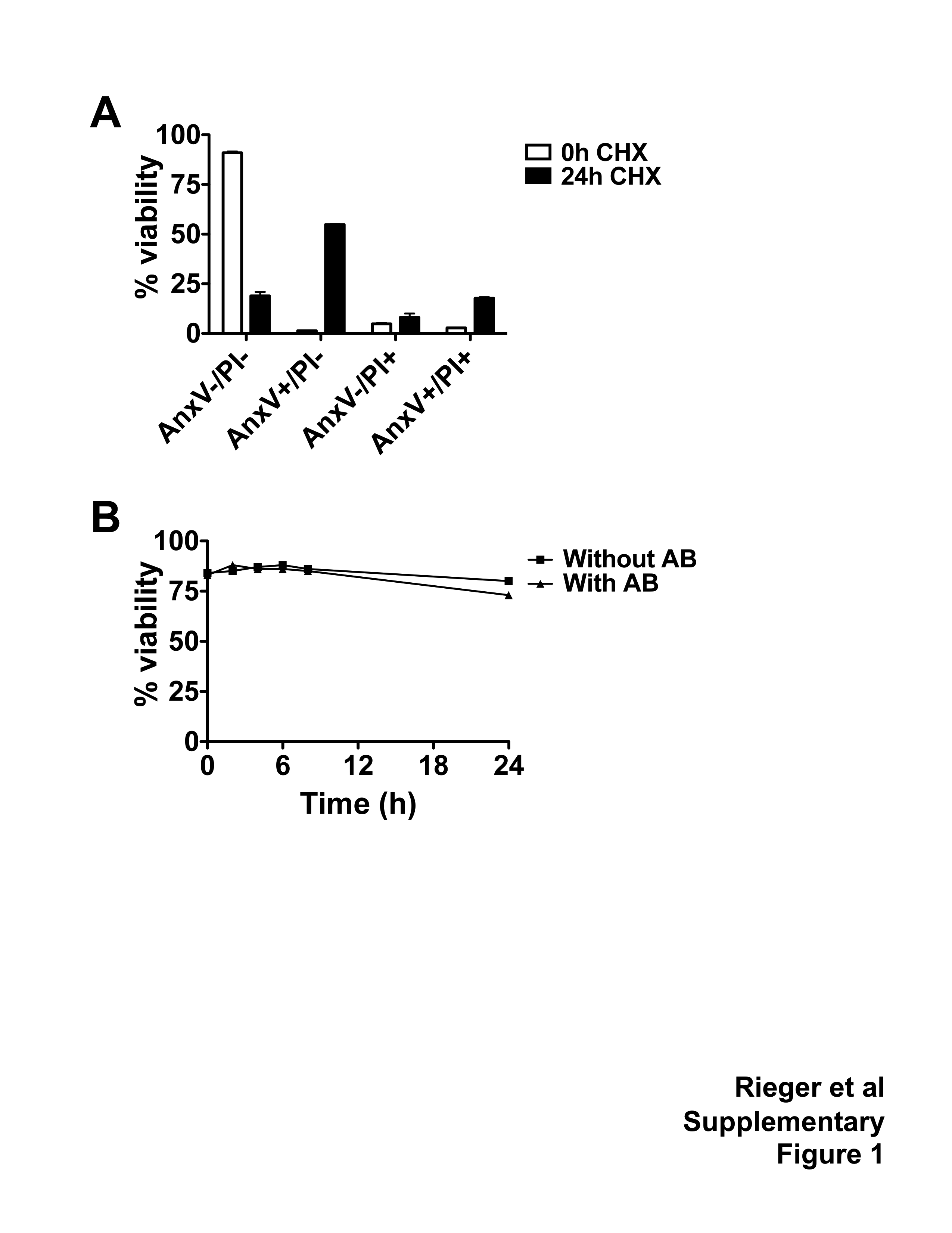

Supplement: Figure S1 — Cycloheximide primarily induces apoptosis in treated cells and can be effectively removed from apoptotic body preparations. (A) Representative experiments show apoptotic bodies generated for treatment of goldfish phagocytes. 3B11 catfish B cells were cultured for 24 h in the presence of 10 μg/mL cycloheximide. Cells were subsequently harvested and stained with Annexin V/propidium iodide to determine cell viability. Cycloheximide treatment primarily induced apoptotic cell death for effective generation of apoptotic bodies, which were then labeled with wheat germ agglutinin-Alexa Fluor 555 overnight. (B) To ensure that apoptotic body preparations did not negatively impact phagocyte viability, apoptotic body preparations were washed three times in 1x PBS−/− to remove remaining cycloheximide and added to goldfish PKM for the times indicated. At these time points, PKM cells were harvested and stained with Annexin V/propidium iodide to assess viability status of these phagocytes. Apoptotic bodies did not induce cell death in PKM cultures. (TIF) [file pone.0047070.s001.tif]

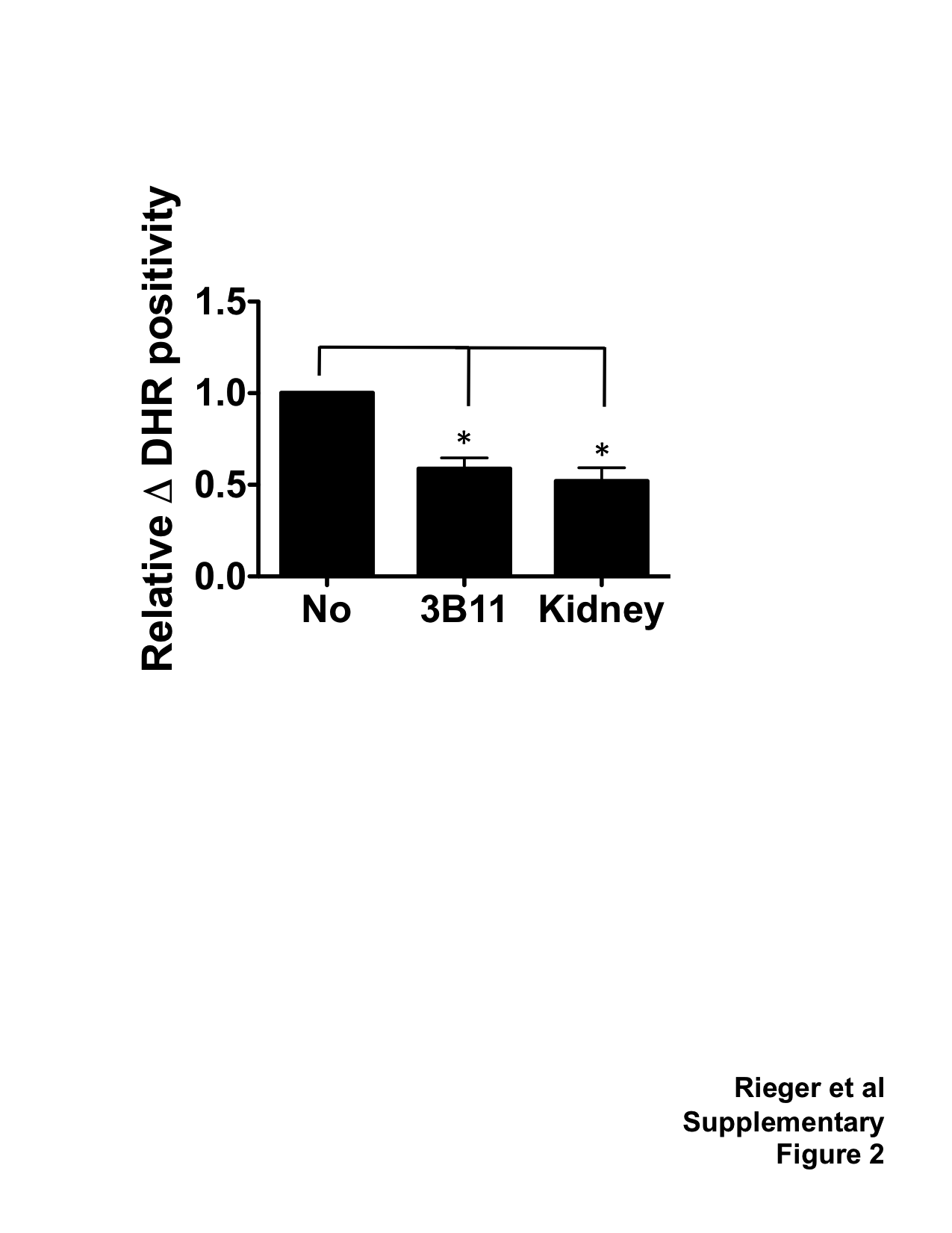

Supplement: Figure S2 — Two distinct sources of apoptotic bodies repress goldfish macrophage respiratory burst to equivalent levels. PKM cultures were incubated with apoptotic bodies derived from 3B11 B cells or goldfish kidney leukocytes. Cells were incubated for 2 h (5∶1, particle: cell ratio) and respiratory burst was analyzed by DHR. There was no significant difference in PKM responses to 3B11-derived or kidney-derived apoptotic bodies. (TIF) [file pone.0047070.s002.tif]

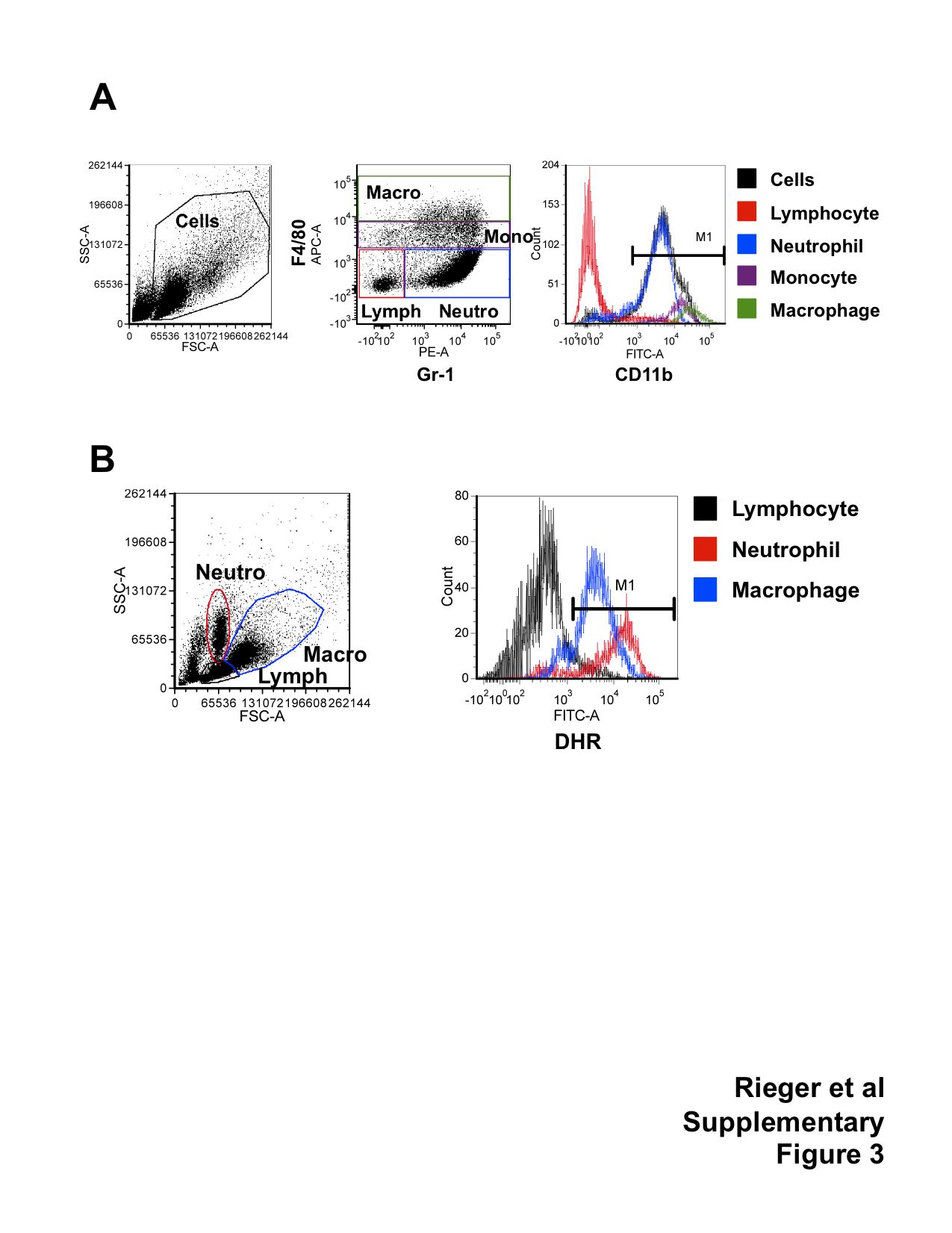

Supplement: Figure S3 — Gating strategy for cell subpopulations isolated from murine peritoneum. (A) Peritoneal cells from C57BL/6 mice were stained with combination of CD11b-FITC/Gr1-PE/F4/80-APC or CD11b-PE-Cy7/CD3-FITC/B220-PE/NK1.1-APC to determine the infiltration of granulocytes (F4/80−/Gr1+/CD11b+), macrophages (F4/80hi/Gr1+/−/CD11b+), monocytes (F4/80lo/Gr1+/−/CD11b+) and lymphocytes (F4/80−/Gr1−). Lymphocyte populations were confirmed to contain T cells (CD11b−/CD3+), B cells (CD11b+/−/B220+) and NK cells (CD11b−/NK1.1+). (B) Murine peritoneal cells were stained with DHR and analyzed using a FACSCanto II flow cytometer. Cell populations were determined based on forward (FSC-A) and side scatted (SSC-A) characteristics. (TIF) [file pone.0047070.s003.tif]

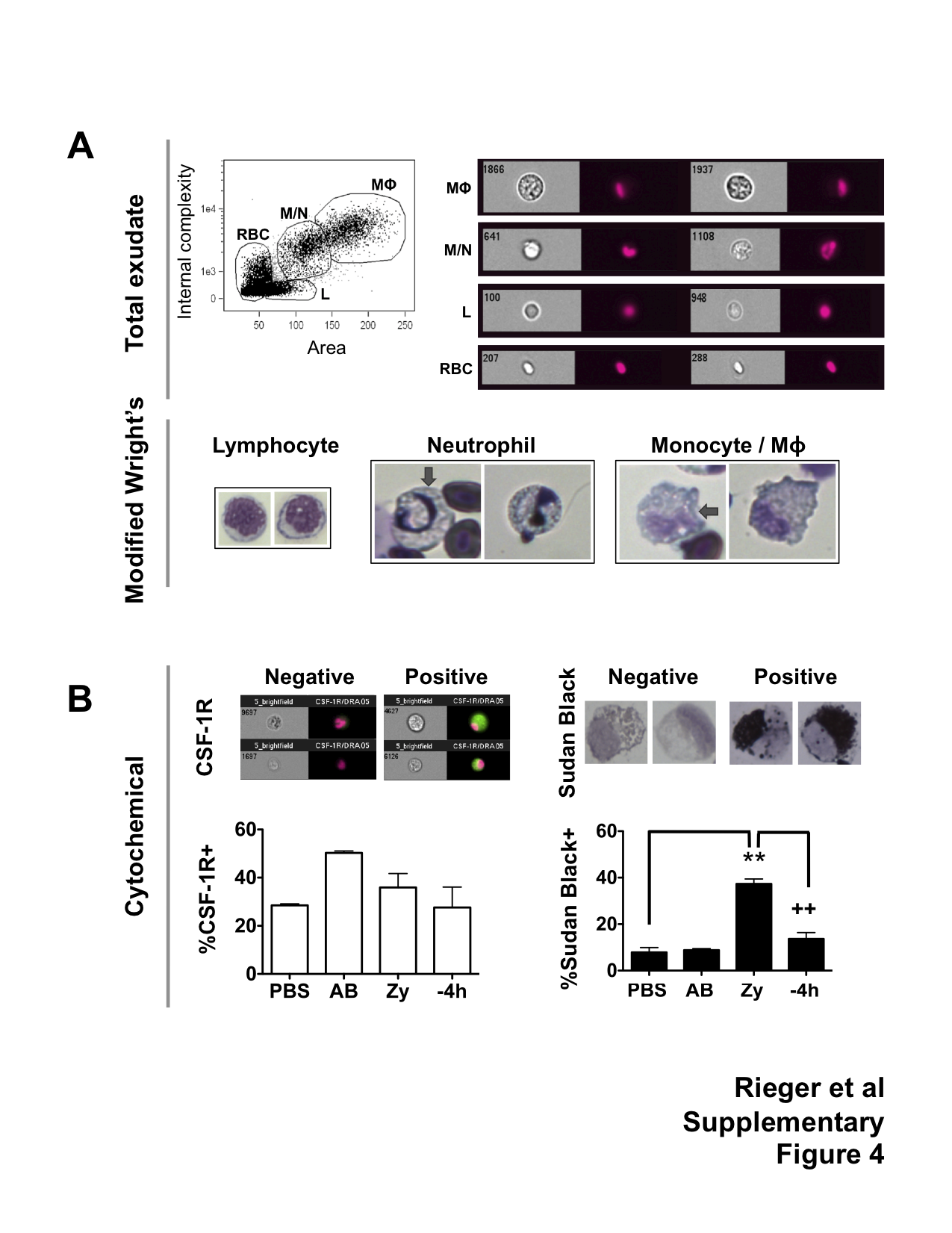

Supplement: Figure S4 — Characterization of goldfish peritoneal myeloid cells. (A) Total peritoneal exudates were analyzed by imaging flow cytometry and four distinct cellular subsets characterized based on area, internal complexity, and morphology. Unlike macrophage and lymphocyte subsets, monocytes and neutrophils could not be subdivided into two distinct populations solely based on these parameters. Modified Wright's stain confirmed the presence of cells with classical lymphocyte, neutrophil, and monocyte/macrophage morphology. (B) To better differentiate between myeloid populations within the goldfish peritoneal exudate, cells were analyzed based on surface CSF-1R expression and Sudan Black cytochemical staining, which denote monocyte/macrophages and neutrophils, respectively. Representative cells stained with anti-CSF-1R antibodies or Sudan Black are shown. Goldfish were injected intraperitoneally with saline, apoptotic bodies (AB; 5×10^6^) or zymosan (Zy; 2.5 mg) and incubated for 24 h. Apoptotic bodies were also pre-injected 4 h (−4 h) before zymosan injections to assess the contributions of kinetics to these responses. For flow cytometry, n = 2; for cytochemical stains, n = 4. * p<0.05 and ** p<0.01 compared to control; ++p<0.01 compared to zymosan. (TIF) [file pone.0047070.s004.tif]

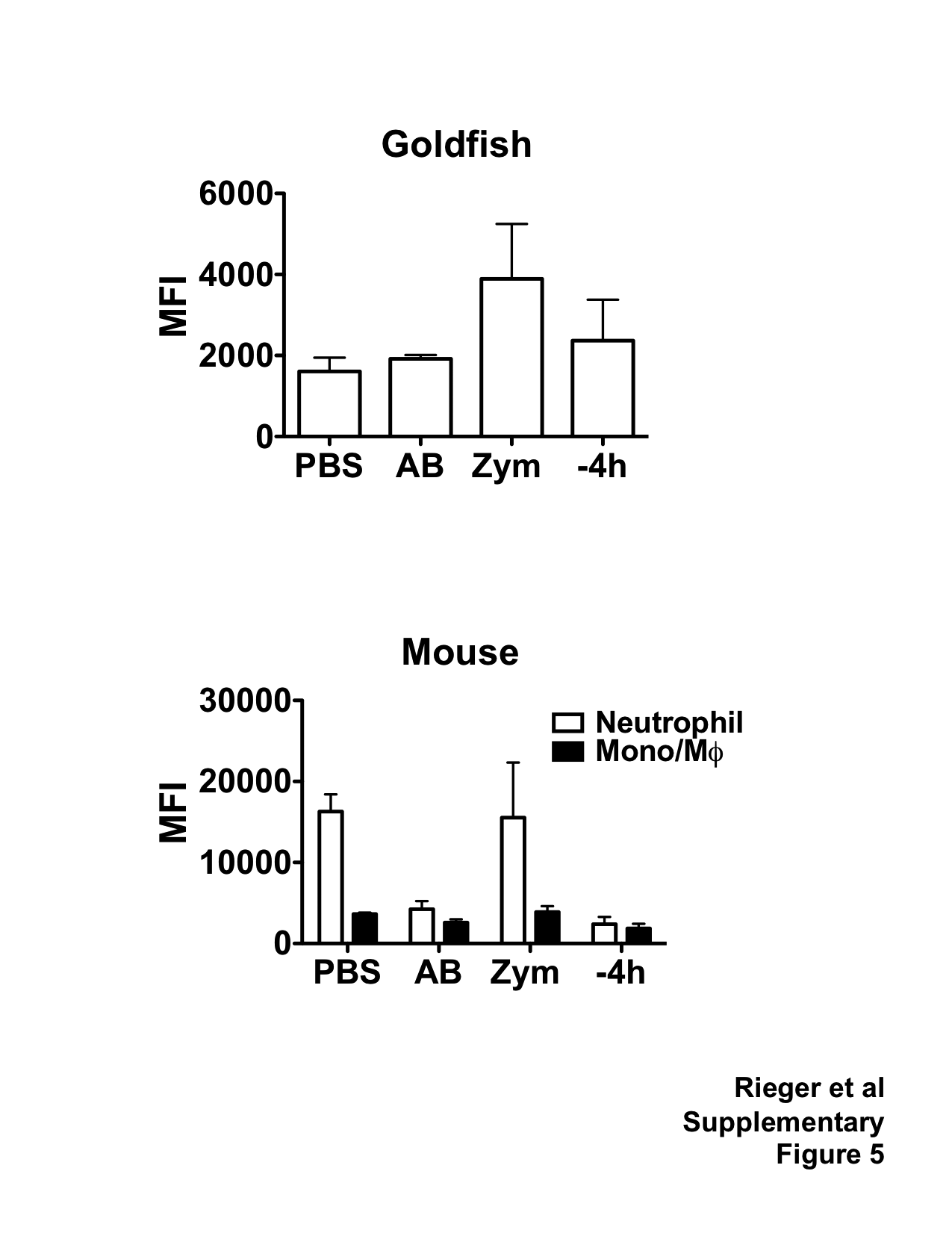

Supplement: Figure S5 — Mean fluorescence intensity of teleost and murine phagocytes. Goldfish and mice were injected intraperitoneally with saline, species-specific apoptotic bodies (5×10^6^) or zymosan (2.5 mg). Apoptotic bodies were also pre-injected 4 h before zymosan injections. Cells from injected animals were harvested by peritoneal lavage and respiratory burst was assayed with DHR in peritoneal cell subpopulations based on forward scatter and side scatter profiles. The mean fluorescence intensity was calculated based on the mean DHR fluorescence in the entire population. For goldfish, myeloid cells are shown. For mice, phagocyte populations were further split into neutrophils and monocyte/macrophages. (TIF) [file pone.0047070.s005.tif]
